# Translation and validation of the Alberta Context Tool for use in Norwegian nursing homes

**DOI:** 10.1371/journal.pone.0258099

**Published:** 2021-10-08

**Authors:** Jannicke Igland, Thomas Potrebny, Bente E. Bendixen, Anne Haugstvedt, Birgitte Espehaug, Kristine B. Titlestad, Birgitte Graverholt

**Affiliations:** 1 Faculty of Health and Social Sciences, Western Norway University of Applied Sciences, Bergen, Norway; 2 Department of Global Public Health and Primary Care, University of Bergen, Bergen, Norway; 3 Faculty of Health Studies, VID Specialized University, Bergen, Norway; University of Tripoli, LIBYA

## Abstract

**Purpose:**

Organizational context is recognized as important for facilitating evidence-based practice and improving patient outcomes. Organizational context is a complex construct to measure and appropriate instruments that can quantify and measure context are needed. The aim of this study was to translate and cross-culturally adapt the Alberta Context Tool (ACT) to Norwegian, and to test the reliability and structural validity among registered nurses (RNs) and licenced practice nurses (LPNs) working in nursing homes.

**Methods:**

This study was a validation study utilizing a cross-sectional design. The sample consisted of n = 956 healthcare personnel from 28 nursing homes from a municipality in Norway. In the first stage, the ACT was translated before being administered in 28 nursing homes. In the second stage, internal consistency and structural validity were explored using Cronbach’s alpha and confirmatory factor analysis.

**Results:**

A rigorous forward-and-back translation process was performed including a team of academics, experts, professional translators and the copyright holders, before an acceptable version of the ACT was piloted and finalized. The Norwegian version of the ACT showed good internal consistency with Chronbachs alpha above .75 for all concepts except for Formal interactions where the alpha was .69. Structural validity was acceptable for both RNs and LPNs with factors loadings more than .4 for most items.

**Conclusions:**

The Norwegian version of the ACT is a valid measure of organizational context in Norwegian nursing homes among RNs and LPNs.

## Introduction

Organizational context is recognized as important in facilitating evidence-based practice (EBP) and improved patient outcomes [1–5]. Organizational context refers to the factors that create a work environment [6–8], such as social interactions, leadership and physical environment [9]. Healthcare organizations that successfully transfer new evidence into practice demonstrate a learning culture that supports the use of knowledge in practice, have leaders who inspire staff to put knowledge to action and have established evaluation through feedback processes [6,10,11]. Interventions that consider contextual factors can address and modify factors within the context, and thus facilitate for evidence-based practice (EBP) and continuously improve safe patient care [6,12]. Consequently, exploring contextual factors specific to the healthcare setting has been highlighted as important to succeed with implementation efforts [13,14]. This is also important in long-term care (LTC), which provides older adults with healthcare and support. Nursing homes are a cornerstone of LTC in several countries [4,15,16] and research on nursing home care has been identified as a high priority for international research [17]. Nevertheless, context is under-investigated in care settings of older adults [10,18]. The scarcity of research on context to guide and support implementation of evidence-based knowledge in the setting of older adults is unfortunate and an area where more research is needed [14].

To address the knowledge gaps in organizational context for nursing homes, it is essential to have a valid tool that measures relevant contextual factors. The Alberta Context Tool (ACT) was developed to determine which elements of context hinder or facilitate successful uptake of research findings, often coined as knowledge translation. ACT measures staff perspectives on modifiable contextual factors relevant to the use of research evidence in healthcare settings [19]. Currently, ACT is utilized in eight countries and is available in six languages [20]. It has been demonstrated that the ACT is a valid tool for measuring context relevant for knowledge translation in healthcare, including nursing homes [20–23]. The aim of this study was to translate and cross-culturally adapt the ACT to Norwegian, and to test the reliability and structural validity of the Norwegian version among registered nurses (RNs) and licenced practice nurses (LPNs) in nursing homes.

## Methods

### Setting

This study was undertaken in nursing homes in one of the larger cities in Norway. Nursing home care in Norway is highly regulated and part of the decentralised responsibility of municipalities to provide long-term care for their citizens. The right to receive care is regulated by the Municipal Health Services Act. Nursing home care services are funded partly by block grants from the state, by tax revenues and by means tested co-payment for long-term residents [24]. Norway holds, compared to other OECD countries, the highest rate of formal care workers in long-term care, counting 12.5 per 100’ population (compared to the OECD average of 4.9 per 100’). Gender distribution for staff in the LTC sector in Norway mirrors the state in other OECD countries, with 90% women or more [25].

This study is part of the integrated knowledge translation project IMPlementation and Action for Knowledge Translation (IMPAKT) [26], with a randomized controlled implementation trial as the main study [27]. The present study was undertaken prior to randomization for the main trial, where a selection of the facilities in this validation study will be invited to participate.

### Design

The ACT questionnaire was translated into Norwegian in accordance with acknowledged methodology [28] and the copyright holders’ manual [29]. To test for reliability and validity we employed a cross-sectional design guided by the COnsensus-based Standards for the selection of health Measurement INstruments (COSMIN) framework [30].

### The Alberta Context Tool

The ACT was developed to assess modifiable and essential elements of context associated with the implementation and uptake of research evidence in practice [19–21]. Its development was based on the construct of context as presented in the Promoting Action on Research Implementation in Health Services (PARIHS) framework [31] and empirical literature reporting contextual factors related to the use of evidence in practice [32,33]. The ACT consists of eight concepts of organizational context with 56 items: *Culture* (six items), *Leadership* (six items), *Evaluation* (six items), *Social capital* (six items), *Informal interactions* (seven items), *Formal interactions* (five items), *Structural and electronic resources* (11 items), and *Organizational slack* (nine items representing the three sub-concepts time, space, human resources) [19] (Concepts in the ACT survey, definitions and examples: S1 Appendix**).** Most items are answered using a five-point Likert scale with the answer alternatives *strongly disagree*, *disagree*, *neither agree nor disagree*, *agree* and *strongly agree*. The exceptions are items in the three concepts measuring *Formal interactions*, *Informal interactions* and *Electronic resources*, where answer alternatives are *never*, *rarely*, *occasionally*, *frequently* and *almost always*. These items were recoded into 0 (never and rarely), 0.5 (occasionally) and 1 (frequently and almost always) and concept scores were calculated as the sum of the recoded items [29]. The range for the concept score varies from 1 to 11, depending on the number of items included in the concept. For the former five concepts the concept scores are obtained as the mean of the Likert scale items with concept scores, ranging from 1 to 5. The recoding was done in accordance with the ACT manual [29]. The ACT has previously been adapted to different healthcare settings and providers [19–23]. We employed the version for nurses in nursing homes.

### Translation and cross-cultural adaptation process

Permission to translate the ACT from English into Norwegian from the copyright holder was obtained and the procedure was performed in line with recommended methodology by the copyright holders [29] and the World Health Organization [28]. We aimed at a conceptual and cultural equivalence, rather than a word-by-word translation.

The ACT was translated to Norwegian independently by two persons fluent in both languages. Both translators had academic backgrounds from EBP and one had a clinical background from nursing homes. A written report from each translator was developed. An agreed-upon version was reached between the forward translators and then presented to a bilingual expert group which consisted of five academics with an EBP background; a professor, an associate professor and three assistant professors. Three of the members had nursing experiences. Two bilingual professional translators, whose native language was English, then conducted a backwards translation to English independently and blinded. They reached agreement upon one common backward translation. The expert group was involved a second time, to provide inputs around conceptual and cultural adaptation, including wording. At this point, it was suggested to include ‘practice development nurse’ as an additional item under the concept *Informal interaction*. Practice development nurses are common in Norwegian healthcare and often hold the responsibility for professional development among care staff. The suggested Norwegian ACT was then sent to the copyright holders, together with the translations and a full description of the process. Correspondence went back and forth until an acceptable version was agreed upon between the expert group and the copyright holders.

To uncover language issues and difficulties in understanding individual items, the Norwegian version of the ACT was pre-tested by six RNs and six LPNs in one nursing home, separate from the validation study sample. All participants completed the questionnaire in paper form while they read aloud the item response options and their own choice of answer. Then the participants were interviewed to elaborate on items or response options that were unclear. The data from the interviews were organized and summarized using a Respondent Problem Matrix (S2 Appendix), a procedure to standardize the cognitive data collected. The procedure is used to identify items that are unclear to respondents [34]. The unclear items were divided into the categories lexical, temporal, logical, inclusion/exclusion and computational problems (terms defined in S2 Appendix). Categories like lexical problems (i.e., not knowing the meanings of words or how to use them) and logical problems (i.e., an item has more than one focus or includes denials, contradictions, tautologies or repetitions) identified the participants’ understanding of an item and whether it measured various aspects of the same characteristic or construct. The participants were asked to suggest alternative wording, and by clarifying the items with high scores (e.g., unclear items), we aimed to strengthen the instrument’s consistency. The results of the pilot were presented to the expert group, whereby a few minor reformulations were decided upon, before the final Norwegian version of ACT was arrived at. These minor reformulations were for example to change words that were considered to be formal, such as “seeks feedback” to “looks for feedback”. The final translation can be found in S3 Appendix.

#### Recruitment and data collection

In Norway, municipality run nursing homes is the major contributor for LTC for older people, although private nursing homes also exist and are important in this care setting. The nursing homes are different in size and location (city/rural) but are administered in the same system and social function, led by the Head of Division of Nursing Homes in the municipality. We invited all 34 nursing homes in a large municipality to participate, of which 28 accepted the invitation. Upon acceptance we established a contact person in each facility to assist us with the recruitment of the facilities’ RNs and LPNs. The number of eligible RNs and LPNs in the nursing homes was provided by the nursing home administration. The ACT questionnaire was self-administered and answered individually on paper. Each nursing home organized the time and place to respond to the questionnaire within working hours. Verbal and written information was provided prior to distribution of the questionnaire. Participation was considered informed consent. Participants were included if they were RNs and LPNs who held a position of at least 25% of full-time equivalent, had worked in the nursing home for at least three months and were able to read and write in Norwegian.

### Evaluation of measurement properties

#### Analysis

Data were analysed using Stata version 15 [35]. The COSMIN checklist was used as a framework to guide the study in choice of sample size and measurement properties [30]. After data entry, 10% of the surveys were control-entered. Demographic characteristics of the study population are described in terms of frequency distributions, percentages, means and standard deviations.

#### Reliability

Internal consistency, as a measure of reliability, was tested by calculating Cronbach’s alpha and item-rest correlation for items within each concept of the instrument. Values of > 0.70 were regarded as acceptable, whereas values of > 0.75 indicated good internal consistency. Internal consistency was also measured using item-total correlations, by correlating each item score with the total score of all the items of the concept, not including the item in question. A positive correlation is seen when items contribute to the total of the scale and a correlation coefficient of >0.3 is considered adequate for items to remain in the scale [19].

Since individual scores of the ATC is commonly aggregated at the unit level and used as a measure of organizational context for an entire unit, we also calculated aggregation statistics in accordance with previous studies [11]. We calculated two different types of intraclass correlation coefficients based on results from oneway random-effects ANOVA with nursing home as grouping variable. ICC(1) was calculated as (BMS—WMS)/(BMS + [K—1] WMS), where BMS is the between-group mean square, WMS is the within-group mean square and K is the average number of respondents per nursing home and calculated as K = (1/[N—1]) (ΣK-[ΣK2 /ΣK]) where N is the number of nursing homes. ICC(2) was calculated as (BMS—WMS)/BMS. ICC(1) is a measure of agreement among the respondents about the mean values on the ACT concepts within each nursing home. ICC(2) is a measure of the reliability of the nursing home mean. Values above 0.10 for ICC(1) and above 0.60 for ICC(2) are considered acceptable [11]. Further unit level aggregation was not possible due to our ethical board’s considerations of the anonymity of respondents.

#### Structural validity

The internal structure of the ACT was assessed using confirmatory factor analysis (CFA). In line with previous validation studies of the ACT [20,21] we estimated three different factor models: Model 1 contained all 10 ACT concepts, Model 2 contained the seven Likert-scaled concepts and Model 3 contained the three count-based ACT concepts. All models were estimated with the variance for each latent variable constrained to 1 and the mean constrained to zero. Latent variables were allowed to correlate with each other. The reported loadings are standardized loadings. We calculated the following fit parameters to assess for model fit: root mean square error of approximation (RMSEA), comparative fit index (CFI), Tucker Lewis index (TLI), standardized root mean square residual (SRMR) and the chi-square test. Good fit for these parameters was set to cut-off values: RMSEA< 0.06, SRMR < 0.8 and CFI > 0.95 [36]. Missing values were handled by choosing full information maximum likelihood (FIML) for missing data as the estimation method for CFA in Stata, under the assumption that missing data are missing at random (MAR) [37]. FIML allows all observations to contribute to the estimation if they do not have missing values on all items. The assumption of MAR is not testable, but we evaluated associations between missing indicator variables (coded with 1 for missing and 0 for non-missing) for each item and the concept score for the concept the item belonged to using logistic regression. There were no significant associations between concept scores and the probability of having a missing value for an item within the concept. Also, the proportion of missing values was low for all items, so any violation of MAR would have to be strong in order to affect the parameter estimates.

In addition to models for the total study population we estimated separate models for RNs and LPNs. We tested for measurement invariance across the two groups for Model 2 with the seven Likert-scaled ACT concepts included with the following procedure: First, we evaluated configural invariance by estimating a common model for RNs and LPNs with stratification on profession, where we allowed loadings to vary freely between professions. We constrained loadings for *Leadership* item 1, variance and mean to be equal to one for RNs and allowed parameters to vary freely for LPNs. Model fit parameters were estimated for the model and SRMR fit statistic was compared between the two professions. Next, we evaluated metric invariance by repeating the model estimation with equal loadings across the two professions as an extra constraint. We performed a likelihood ratio chi-square test by comparing the measurement invariance model to the configural invariance model. A significant chi-square test indicated different loadings across professions. Scalar invariance was evaluated by adding an additional constraint on equal intercepts for all items across the two professions and the model was compared with the configural invariance model by applying a likelihood ratio chi-square test. Because Model 3 showed poor fit both on RNs and LPNs we did not test for measurement invariance across professions for the count-based ACT concepts.

### Ethical considerations

The Norwegian Centre for Research Data (NSD), the data protection official for research in Norwegian universities, approved the study (reference number 49918). Data are stored in a secure server with access control.

## Results

### Translation and cross-cultural adaptation

The forward–backward translation was repeated three times before arriving at an acceptable version. The pilot participants confirmed face validity. The expert panel assessed content validity and found the questionnaire, questions and rating scale clinically reasonable and relevant for the setting of Norwegian nursing homes. We considered the translation process to result in a linguistic, semantic and contextual equality between the Norwegian and the original versions of the ACT.

### Sample characteristics

Among the 28 nursing homes who accepted our invitation to participate, there were a total of 1814 eligible participants. Among the 1014 (56%) individuals who completed the survey 58 were excluded because they did not fulfil the inclusion criteria. A total number of 956 respondents were included and analysed. The response rate ranged from 47% to 76% between facilities. Most of the respondents were female (92%), half of them were between 40 and 59 years old (51%), 59% were LPNs and the participants had worked in the present nursing home for an average of 10 years (SD ± 7.8). Demographic characteristics of the respondents are shown in Table 1. We regard this sample as representative for nursing homes facilities in Norway in terms of organizational characteristics such as ownership model, size and staffing.

**Table 1:** Demographic characteristics of the RNs and LPNs (N = 956).

		Total sample	Registered Nurse	Licensed Practice Nurse	p-value
**n**		956	391	565	
**Sex, n (%)**					
	Male	70 (7.4)	22 (5.7)	48 (8.5)	
	Female	878 (92.6)	363 (94.3)	515 (91.5)	0.10
**Age, n (%)**					
	20–39	335 (35.3)	154 (39.6)	181 (32.3)	
	40–59	480 (50.5)	208 (53.5)	272 (48.5)	
	60+	135 (14.2)	27 (6.9)	108 (19.3)	<0.001
**Years since completed education, mean (SD)**		14.9 (11.3)	15.1 (11.2)	14.8 (11.4)	0.68
**Years worked at current nursing home, mean (SD)**		9.8 (7.9)	7.7 (6.7)	11.3 (8.3)	<0.001
**Job content**					
	Full time	511 (54.0)	252 (64.8)	259 (46.4)	
	Part time	436 (46.0)	137 (35.2)	299 (53.6)	<0.001
**Type of shift**					
	Daytime	719 (75.9)	312 (80.6)	407 (72.7)	
	Evening	128 (13.5)	31 (8.0)	97 (17.3)	
	Night	100 (10.6)	44 (11.4)	56 (10.0)	<0.001
**Mother tongue**					
	Norwegian	702 (73.7)	281 (72.2)	421 (74.7)	
	Other	251 (26.3)	108 (27.8)	143 (25.4)	0.407

### Evaluation of measurement properties

#### Reliability

For the ACT concepts the missing data on items ranged from 0.6% to 4.9% and 641 questionnaires had no missing data on any of the ACT items. Descriptive statistics for the items are shown in Table 2.

**Table 2:** ACT item statistics.

Concept	Item	n	N missing (%)	Mean (SD)	Min-max
*Leadership*	Looks for feedback	940	16 (1.7)	3.6 (1.0)	1–5
	Focuses on successes	942	14 (1.5)	3.7 (1.09	1–5
	Calmly handles stress	947	9 (0.9)	3.9 (0.9)	1–5
	Listens, acknowledges, responds	943	13 (1.4)	3.9 (1.0)	1–5
	Actively mentors and coaches	935	21 (2.2)	3.7 (0.9)	1–5
	Resolves conflicts	942	14 (1.5)	3.6 (1.0)	1–5
*Culture*	Receive recognition	941	15 (1.6)	3.7 (0.9)	1–5
	Control over work	948	8 (0.8)	4.2 (0.6)	1–5
	Organization balances	930	26 (2.7)	3.5 (0.8)	1–5
	Professional development	942	14 (1.5)	3.6 (0.9)	1–5
	Clear on what patients want	949	7 (0.7)	4.4 (0.6)	1–5
	Supportive work group	950	6 (0.6)	4.2 (0.8)	1–5
*Evaluation*	Routinely receive information	942	14 (1.5)	3.6 (1.0)	1–5
	Discusses data informally	940	16 (1.7)	3.5 (0.9)	1–5
	Formal process	942	14 (1.5)	3.3 (1.0)	1–5
	Formulates action plans	935	21 (2.2)	3.6 (0.9)	1–5
	Monitors our performance	937	19 (2.0)	3.4 (0.9)	1–5
	Compares our performance	936	20 (2.1)	3.0 (0.9)	1–5
*Social Capital*	Share information with others	946	10 (1.1)	4.2 (0.6)	1–5
	Observations are taken seriously	943	13 (1.4)	4.1 (0.7)	1–5
	Information is shared	941	15 (1.6)	3.6 (0.9)	1–5
	Comfortable talking in authority	941	15 (1.6)	4.1 (0.7)	1–5
	Aim is to help others	937	19 (2.0)	4.1 (0.7)	1–5
	Group participation is valued	931	25 (2.6)	4.0 (0.7)	1–5
*Slack–Staff*	Get the necessary work done	944	12 (1.3)	2.8 (1.2)	1–5
	Deliver best possible care	942	14 (1.5)	2.5 (1.1)	1–5
	Give patients a good day	940	16 (1.7)	2.6 (1.0)	1–5
*Slack–Space*	Adequate space	936	20 (2.1)	3.6 (1.1)	1–5
	Private space	940	16 (1.7)	3.5 (1.2)	1–5
	Use of private space	945	11 (1.2)	2.5 (1.5)	1–5
*Slack–Time*	Do something extra for patients	938	18 (1.9)	2.3 (0.9)	1–5
	Talk about plan of care	939	17 (1.8)	2.3 (0.9)	1–5
	Look something up	933	23 (2.4)	2.2 (1.0)	1–5
	Talk about new clinical knowledge	932	24 (2.5)	2.0 (1.0)	1–5
*Informal Interactions*	Nurse	937	19 (2.0)	3.2 (1.3)	1–5
	Physicians	935	21 (2.2)	2.1 (1.1)	1–5
	Assistant	920	36 (3.8)	2.9 (1.3)	1–5
	Allied health care provider	935	21 (2.2)	3.4 (1.2)	1–5
	Other healthcare providers	919	37 (3.9)	1.9 (1.1)	1–5
	Research nurse or coordinator	928	28 (2.9)	1.1 (0.4)	1–5
	Clinical educator/instructor	931	25 (2.6)	1.3 (0.6)	1–5
	Quality improvement representative	919	37 (3.9)	1.4 (0.7)	1–5
	Champion	918	38 (4.0)	1.2 (0.5)	1–5
	“Hallway talk”	931	25 (2.6)	2.8 (1.4)	1–5
	Informal bedside teaching	922	34 (3.6)	1.0 (1.1)	1–5
	Practice Development Nurse	935	21 (2.2)	1.7 (0.9)	1–5
*Formal Interactions*	Team meetings	936	20 (2.1)	2.0 (1.2)	1–5
	Interdisciplinary meetings about patients	936	21 (2.2)	2.0 (1.2)	1–5
	Family conferences/ next of kin meetings	935	21 (2.2)	1.6 (0.7)	1–5
	Education out of workplace	945	11 (1.2)	1.8 (0.7)	1–5
*Structural/Electronic Resources*	Library	939	17 (1.8)	1.1 (0.4)	1–5
	Health library (online)	928	28 (2.9)	1.4 (0.7)	1–5
	Textbooks	930	26 (2.7)	1.5 (0.8)	1–5
	Journals (printed/online)	920	36 (3.8)	1.8 (1.0)	1–5
	Notice boards	926	30 (3.1)	2.7 (1.3)	1–5
	Policies and procedures	916	40 (4.2)	2.3 (1.3)	1–5
	Clinical practice guidelines	909	47 (4.9)	2.3 (1.2)	1–5
	Professional procedures	933	23 (2.4)	2.6 (1.2)	1–5
	Computerized decision support	918	28 (4.0)	2.4 (1.5)	1–5
	Reminders (ex. via e-mail)	927	29 (3.0)	2.2 (1.3)	1–5
	Websites	930	26 (2.7)	2.3 (1.2)	1–5
	Internal training/workshops at work	933	23 (2.4)	1.7 (0.8)	1–5

Of the 10 ACT concepts, nine had alpha coefficients above 0.75, which indicates good internal consistency. One exception was the concept *Formal interactions* which had an alpha of 0.69.

All items within concepts related well to the total test score of the concept and most of the items had item-total correlations of > 0.4, which indicate good internal consistency. The concepts of *Leadership*, *Feedback*, *Staff* and *Time* revealed the highest item-total correlations with maximum coefficients ranging from 0.82 to 0.92. The lowest item-total correlation was observed for item six for the concept of *Informal interactions* with an item-total correlation of 0.33. Table 3 shows the reliability measurements of the ACT concepts. All ICC(1) values were greater than 0 and two were above 0.1, indicating some degree of perceptual agreement among the respondents within a nursing home [11]. ICC(2)-values was above 0.60 for eight out of eleven concepts indicating reliable measurements of the ACT concepts when individual responses were aggregated at the nursing home level [11].

**Table 3:** Reliability measures for the concepts in the ACT.

Concepts	ACT score, mean (SD)	Cronbach`s alpha	Item-total correlation, (min-max)	ICC(1)^a^	ICC(2)^b^
*Leadership* (range 1–5)	3.73 (0.77)	0.89	0.71–0.84	0.06	0.67
*Culture* (range 1–5)	3.95 (0.52)	0.76	0.53–0.76	0.06	0.66
*Feedback* (range1-5)	3.40 (0.75)	0.88	0.70–0.86	0.06	0.67
*Formal interactions* (range 0–4)	0.41 (0.75)	0.69	0.51–0.84	0.05	0.65
*Informal interactions* (range 0–12)	2.54 (2.12)	0.83	0.33–0.78	0.03	0.53
*Social capital* (range 1–5)	4.01 (0.53)	0.79	0.65–0.76	0.02	0.45
*Structural/Electronic resources* (range 0–12)	2.22 (2.25)	0.81	0.43–0.69	0.02	0.39
*Organizational slack*				0.11	0.81
*Space* (range 1–5)	3.11 (1.10)	0.77	0.61–0.92	0.12	0.82
*Staff* (range 1–5)	2.61 (0.99)	0.88	0.88–0.92	0.02	0.97
*Time* (range 1–5)	2.19 (0.78)	0.82	0.78–0.82	0.06	0.67

^a^ ICC(1) = (BMS—WMS)/(BMS + [K—1] WMS), where BMS is the between-group mean square, WMS is the within-group mean square and K is the average number of respondents per nursing home. K was calculated as K = (1/[N—1]) (ΣK-[ΣK2 /ΣK]) where N is the number of nursing homes.

^b^ ICC(2) = (BMS—WMS)/BMS.

### Confirmatory factor analysis

We assessed three factor models for the total study population (Table 4).

**Table 4:** Standardized factor loadings and model fit parameters for confirmatory factor analysis (N = 956).

		Factor Loadings		
Concept	Item ^a^	Model 1	Model 2	Model 3
*Leadership*	Looks for feedback	0.676	0.679	
	Focuses on successes	0.593	0.611	
	Calmly handles stress	0.761	0.757	
	Listens, acknowledges, responds	0.829	0.824	
	Actively mentors and coaches	0.836	0.830	
	Resolves conflicts	0.836	0.824	
*Culture*	Receive recognition	0.715	0.706	
	Control over work	0.395	0.421	
	Organization balances	0.648	0.641	
	Professional development	0.668	0.660	
	Clear on what patients want	0.531	0.543	
	Supportive work group	0.564	0.569	
*Evaluation*	Routinely receive information	0.757	0.744	
	Discusses data informally	0.659	0.657	
	Formal process	0.841	0.845	
	Formulates action plans	0.823	0.821	
	Monitors our performance	0.827	0.830	
	Compares our performance	0.626	0.624	
*Social capital*	Share information with others	0.691	0.683	
	Observations are taken seriously	0.703	0.695	
	Information is shared	0.464	0.474	
	Comfortable talking in authority	0.645	0.654	
	Aim is to help others	0.572	0.592	
	Group participation is valued	0.753	0.742	
*Slack–Staff*	Get the *necessary* work done	0.746	0.742	
	Deliver best possible care	0.914	0.909	
	Give patients a good day	0.867	0.883	
*Slack–Space*	Adequate space	0.595	0.605	
	Private space	0.932	0.939	
	Use of private space	0.674	0.664	
*Slack–Time*	Do something extra for patients	0.677	0.687	
	Talk about plan of care	0.761	0.735	
	Look something up	0.722	0.723	
	Talk about new clinical knowledge	0.760	0.750	
*Informal interactions*	Nurse	0.815		0.816
	Physicians	0.615		0.603
	Assistant	0.787		0.792
	Allied healthcare provider	0.855		0.861
	Other healthcare providers	0.549		0.527
	Research nurse or coordinator	0.208		0.204
	Clinical educator/instructor	0.320		0.331
	Quality improvement representative	0.440		0.431
	Champion	0.289		0.281
	Hallway talk	0.457		0.468
	Informal bedside teaching	0.426		0.433
	Practice Development Nurse	0.469		0.468
*Formal interactions*	Team meetings	0.715		0.734
	Interdisciplinary meetings about patients	0.832		0.830
	Family conferences/ next of kin meetings	0.554		0.580
	Education out of workplace	0.333		0.315
*Structural/electronic resources*	Library	0.112		0.115
	Health library (online)	0.275		0.287
	Textbooks	0.406		0.417
	Journals (printed/online)	0.504		0.499
	Notice boards	0.447		0.464
	Policies and procedures	0.666		0.672
	Clinical practice guidelines	0.752		0.772
	Professional procedures	0.741		0.773
	Computerized decision support	0.475		0.456
	Reminders (ex. via e-mail)	0.543		0.533
	Websites	0.560		0.531
	Internal training/workshops at work	0.437		0.431
Model-Data Fit: x2 (p-value)		4912.151 (< .001)	1435.585 (< .001)	2531.263 (< .001)
		df = 1784	df = 506	df = 347
RMSEA ^b^		0.051	0.048	0.092
SRMR ^c^		0.062	0.047	0.090
CFI ^d^		0.825	0.927	0.699

^a^ Horizontal lines separate factors within each model, i.e., there are 10 factors in Model 1, seven in Model 2 and three in Model 3.

^b^ RMSEA = root mean square error of approximation.

^c^ SRMR = standardized root mean square residual.

^d^ CFI = comparative fit index.

In Model 1, all the ACT items were included and each item was loaded on its corresponding ACT concept. For the concept *Informal interactions*, asking ‘In the ***last typical MONTH***, how often did you have a ***patient care related discussion*** with individuals or groups of people in the following roles or situations?’, items six (‘Quality improvement representative/specialist’), seven (‘Any clinical educator/instructor’) and nine (‘Someone who *champions* research in practice’), and the concept *Structural and electronic resources*, asking ‘In the ***last typical MONTH***, how often did you use the following while ***at work***?*’*, item one (‘a library’) showed standardized loadings <0.4 in Model 1. Model fit parameters showed acceptable fit for the parameters RMSEA (0.051) and SRMR (0.061), but CFI was only 0.816 and the chi-square test for deviation from good fit was significant (p<0.001). When items six, seven and nine for *Informal interactions* and item one for Electronic resources were excluded from Model 1, there was an improvement in model fit for RMSEA (0.048), SRMR (0.057) and CFI (0.858).

When splitting Model 1 into one model for *scaled* concepts of the ACT (seven concepts) and one model for *non-scaled* concepts (three concepts) in Model 2 and Model 3, we observed good fit for Model 2 while model fit for Model 3 was poor.

Factor loadings and model fit parameters for Model 1 assessed separately for healthcare workers, RNs and LPNs, are reported in S4 Appendix. Standardized loadings were >0.4 for most items for both RNs and LPNs. Both groups showed acceptable model fit for RMSEA and SRMR, but CFI was <0.90. The chi-square test for deviation from good fit was also significant. Model 2 showed good fit in both groups for RMSEA, SRMR and CFI while Model 3 showed poor fit in both groups.

In evaluation of measurement invariance for Model 2, the configural invariance model showed good fit, with almost identical SRMR for RNs and LPNs (0.052 versus 0.053) (Table 5).

**Table 5:** Test of measurement invariance between RNs and LPNs for the seven scale-based concepts of the ACT.

	Configural invariance model	Metric invariance model	Scalar invariance model
N	815	815	815
RMSEA	0.049	0.049	0.050
CFI	0.922	0.921	0.915
SRMR	0.052	0.060	0.061
SRMR- RNs	0.052	0.063	0.064
SRMR- LPNs	0.051	0.057	0.058
X^2^	1999.9 (<0.001)	2052.3 (<0.001)	2170.4 (<0.001)
df	1012	1045	1078
$\chi_{diff}^{2}$		52.5	170.5
df		33	66
p-value		0.017	<0.001

We therefore conclude that the factor structure in Model 2 fits equally well for RNs and LPNs. The measurement invariance model with constrained loadings also showed acceptable fit, but with slightly weaker fit parameters compared to the configural invariance model. The chi-square test comparing the two models was significant (p = 0.017), indicating that a model with equal loadings for the two professions does not fit the data as well as a model where loadings are allowed to vary freely. The model with scalar invariance in addition to metric invariance showed similar model fit as the scalar invariance model and the chi-square test comparing this model to the configural invariance model was significant (p<0.001), indicating a difference in intercept across professions.

## Discussion

Organizational context has been shown to have a significant impact on successful knowledge translation in healthcare. A reliable and valid tool that measures modifiable contextual factors relevant to research use is thus important for both researchers and care providers. In this Norwegian cross-cultural translation and validation study of the ACT, we investigated reliability and structural validity among 956 RNs and LPNs working in 28 nursing homes in a large municipality. The ACT showed acceptable validity both in terms of reliability and structural validity, measured using confirmatory factor analyses. The English version of the ACT has previously been thoroughly validated by the tool developers and others, in different healthcare settings [19–21,38]. Translated versions of the ACT have also been validated in Swedish [22] and German studies [23]. These studies tested the ACT tool in nursing home populations, but only the German study did an assessment of the psychometric properties. Our findings support the use of the ACT in other languages and healthcare contexts after rigorous translation procedures and the use among healthcare personnel in nursing homes. This validation study indicates that the Norwegian ACT can be recommended for further use in nursing homes, but modifications of some of the concept items can be considered.

### Cross-cultural translation

Cross-culturally translating and validating a tool is an extensive but necessary process. The translation process of the ACT questionnaire into Norwegian relied on an extensive and iterative process that involved researchers, translators, the copyright holders and an expert group. English and Norwegian languages differ in terms of richness and synonyms. For this reason, we were not always able to find a verbatim equivalent, but instead had to reach consensus on semantic equivalence. Where possible, we attempted to use the everyday language used in nursing homes and used terms based on feedback from pilot participants and participants in the expert group with clinical expertise. The results from the analyses of the data from the cognitive interviews lead to modifications on item wording. One strength of this study is therefore that the items are adapted to the Norwegian context through a cross-cultural adaptation translation process.

### Reliability

All concepts except *Formal interactions* showed very good internal consistency measured in terms of Cronbach’s alpha. For *Formal interactions* Cronbach alpha was 0.69, which is slightly below the recommendation of >0.7. The same concept has been shown to have Cronbach alpha < 0.7 in three other validation studies [20,21,23]. The questions within this concept have been designed to measure different types of formal interactions and are intended to be non-redundant. The items are therefore not necessarily meant to be highly correlated. Previous validation studies have attributed the low alpha to this non-redundancy. The principle of non-redundancy has also been applied in the selection of items within the other nine concepts, but the weak correlation between items was only present for one concept in our study. For the other two translated versions of the ACT, the German and the Swedish, alpha was > 0.7 standard in 10 out of 13 concepts [23] and in five out of eight concepts [22].

In our study population, the concept score for *Formal interactions* was also low with an average (SD) of 0.41 (0.75) on a scale with 4 as the maximum possible value. The percentage who answered ‘never (0)’ or ‘rarely (1–5 times)’ varied between 80 and 95% for the four items and less than 2% answered ‘frequently (11–15 times)’ or ‘almost always (16 times or more)’ on the two questions about how often they have attended meetings with relatives the last month and courses outside the facility the last year. It can be questioned whether the numbers of times listed next to each category in the questionnaire were suitable. In most Norwegian nursing homes, it is very rare to attend courses outside the facility several times a year or have formal meetings with relatives several times a month.

For the concept *Informal interactions*, item six (‘How often do you interact with people in the following roles or positions regarding a residents nursing and care needs–Research Nurse’) showed poor item-total correlation (0.33). Almost all (94%) of the participants answered ‘never’ on this question. At this point in time, no nursing homes have research nurses. This was acknowledged during the translation process, but we decided to keep the item as this may change. ICC values measuring agreement between individual respondents within nursing homes were lower than ideal for most ACT-concepts, indicating substantial variation in perception of the organizational context within nursing homes. We are not able to take into account that many of the nursing homes have separate care units, which may make it less appropriate to aggregate scores at the nursing home level. This is also in accordance with Estabrooks et al [11] who found lower ICC-values when aggregating to the nursing home level, compared to the unit level. We did however find high ICC-values for most of the ACT concepts, indicating high reliability for nursing home means in our study. It is therefore still meaningful to compare aggregate means between nursing homes.

### Structural validity

In line with previous validation studies of the ACT [21,23,38], we used three factor models: one for all items, one for items included in the seven scale-based concepts and one for items included in the three count-based concepts, as recommended by the copyright holders [23]. Similar to the previous validation studies, we found improvement in model fit when excluding the count-based items. Also, the model including only count-based items showed poor model fit for all model fit indices. There are several possible reasons for lack of fit for the count-based items. First of all, the distribution for these items appears non-normal, which is one of the assumptions underlying the FIML approach. Second, items included in the count-based concepts are not expected to be highly correlated. This is especially the case for *Structural and electronic resources*. People who have access to one of the resources do not necessarily have access to other resources on the list. Third, respondents generally scored low on items for the three count-based concepts, especially for items within *Formal interactions*. Limited variation in item responses can cause poor model fit.

Even though most model fit indices showed acceptable model fit both for the total model with all concepts and for the model with the seven scale-based concepts, the overall chi-square fit showed significant deviation from good fit. The same lack of fit has been found in all other psychometric assessments of the ACT, both the original version [21] and a translated version [23]. Since the included items were specifically chosen with the purpose of measuring similar, but not identical features of the concepts, we did not expect to find very good model fit in terms of fit indices. The factor model assumes that item variation within a concept has a common cause, which is not necessarily true for the ACT. High factor loadings, Cronbach alpha and item-total statistics did however confirm that it is reasonable to group items into 10 separate concepts.

In the current study the same questionnaire was used for both RNs and LPNs. Analyses of measurement invariance confirmed that the factor structure with 10 concepts was equally valid for both professional groups, but loadings differed between professions, indicating that not all items were equally strongly related to the concept for both professions. Because of this, item scores can be expected to vary between the RNs and LPNs [36]. This should be kept in mind when comparing scores for the various ACT concepts between RNs and LPNs. An observed difference in mean scores does not necessarily reflect a difference in perception of context. The difference could also be caused by a difference in how the items relate to contextual factors for RNs and LPNs.

### Strengths and limitations

The sample size of 956 respondents from 28 nursing homes can be regarded as an appropriate and strong sample. The 28 nursing homes who participated represent a selection of facilities with a wide range of organizational contexts, including differences in size, personnel-situation and leadership-situation, within one municipality in Norway. Within our sample 26% had a mother tongue other than Norwegian and 92% were female, which accurately represent the true population of healthcare workers in Norway [25]. Further analysis of participants characteristics (such as ethnicity or country of origin) could inform this research, however, due to restrictions from the ethical board, additional participant characteristics were not collected.

According to the COSMIN checklist [30] and other guides for psychometric validation of scales, it is recommended to investigate correlation with other instruments when evaluating structural validity. In similar studies [20,39] researchers have correlated concepts scores for the ACT with measures of research utilization and found significant correlations for all concepts. It could have been of interest to investigate if this correlation is also present in Norwegian nursing homes. If the ACT is going to be used to measure readiness for implementation of new practice further studies is needed in order to investigate if ACT is associated with research utilization in a Norwegian context.

The COSMIN checklist recommends that test-retest should be performed as part of evaluation of the instruments’ reliability, however, test-retest often has challenges relating to attrition. The current study originally planned to include a test-retest validation. It was, however, difficult to get the staff at the nursing home to prioritize a second survey and only 55 participated. Among these, only 28 respondents managed to include the same respondent ID on the questionnaire as in the first round, which made it impossible to link questionnaires for the remaining 27. We do also know that one of the nursing homes who participated in the retest had changes in leadership and structure of the care units between test and retest. Due to the aforementioned issues, we were unfortunately not able to conduct the retest as planned.

## Conclusion

The Norwegian version of the ACT is a sufficiently valid instrument for measuring organizational context in Norwegian nursing homes, both in terms of reliability and structural validity. The Norwegian ACT was shown to be equally valid for RNs and LPNs. The findings from this study further support previous research and indicate that the ACT is an appropriate instrument for measuring organizational context within the healthcare setting in a wide variety of comparative healthcare contexts.

## Supporting information

S1 Appendix Concepts in the ACT survey, definitions, and examples. (DOCX)

S2 Appendix The problem respond matrix. (DOCX)

S3 Appendix ACT translation. (DOCX)

S4 Appendix Standardized factor loadings and model-data fit. (DOCX)

S1 Dataset ACT dataset. (XLS)
