# Supplementary material for: Translation and validation of the Alberta Context Tool for use in Norwegian nursing homes
Source: PLoS One. 2021 Oct 8;16(10):e0258099. doi: 10.1371/journal.pone.0258099 (PMC8500415; doi:10.1371/journal.pone.0258099)
Supplement: S1 Appendix — (DOCX) [file pone.0258099.s001.docx]

**S1 Appendix 1. Concepts in the ACT survey, definitions, and examples.**

| **Concept** | **Definition** | **Hypothesis** | **Sample item** |
| --- | --- | --- | --- |
| *Leadership* 1 | The actions of formal leaders in an organization (unit) to influence change and excellence in practice, items generally reflect emotionally intelligent leadership | **H1:** Care providers who perceive more positive (emotionally intelligent) unit leadership report higher research use | The leader calmly handles stressful situations |
| *Culture* 1 | The way that “we do things” in our organizations and work units, items generally reflect a supportive work culture | **H2:** Care providers who perceive a more positive unit culture report higher research use | My organization effectively balances best practice and productivity |
| *Evaluation* 1 | The process of using data to assess group/team performance and to achieve outcomes in organizations or units | **H3:** Care providers who perceive a larger number of unit feedback mechanisms report higher research use | Our team routinely monitors our performance with respect to action plans |
| *Social capital* 1 | The stock of active connections among people. These connections are of three types: bonding, bridging, and linking | **H4:** Care providers who perceive more positive unit social capital activities report higher research use | People in the group share information with others in the group |
| *Formal interactions* 2 | Formal exchanges that occur between individuals working within an organization (unit) through scheduled activities that can promote the transfer of knowledge | **H5:** Care providers who perceive a larger number of formal unit interactions report higher research use | How often do these activities occur?  -Team meetings |
| *Informal interactions* 2 | Informal exchanges that occur between individuals working within an organization (unit) that can promote the transfer of knowledge | **H6:** Care providers who perceive a larger number of informal unit interactions report higher research use | How often do you interact with people in the following roles or positions?  -Someone who champions research and its use in practice |
| *Structural/Electronic resources* 3 | The structural and electronic elements of an organization (unit) that facilitate the ability to assess and use knowledge | **H7:** Care providers who perceive a larger number of unit structural and electronic resources report higher research use | How often do you use/attend the following?  -Notice Boards |
| *Organizational Slack* | The cushion of actual or potential resources which allows an organization (unit) to adapt successfully to internal pressures for adjustments or to external pressure for changes |  |  |
| *Staff* 1 |  | **H8:** Care providers who perceive sufficient unit staffing levels report higher research use | Enough staff to deliver quality care |
| *Space* 2 |  | **H9:** Care providers who perceive having sufficient time on their unit report higher research use | Use of designated space |
| *Time* 1 |  | **H10:** Care providers who perceive having sufficient space on their unit report higher research use |  |

1 = Scale: 1-strongly disagree; 2-disagree; 3-neither agree or disagree; 4-agree; 5-strongly agree

2 = Scale: 1-never; 2-rarely; 3-ocasionally; 4-frequently; 5-almost always

3 = Scale: 1-never; 2-rarely; 3-ocasionally; 4-frequently; 5-almost always; 6-not accessible
