# Supplementary figures and images for: Translation and validation of the Alberta Context Tool for use in Norwegian nursing homes
Source: PLoS One. 2021 Oct 8;16(10):e0258099. doi: 10.1371/journal.pone.0258099 (PMC8500415; doi:10.1371/journal.pone.0258099)

**S2 Appendix 2. The Problem Respond Matrix.**

**
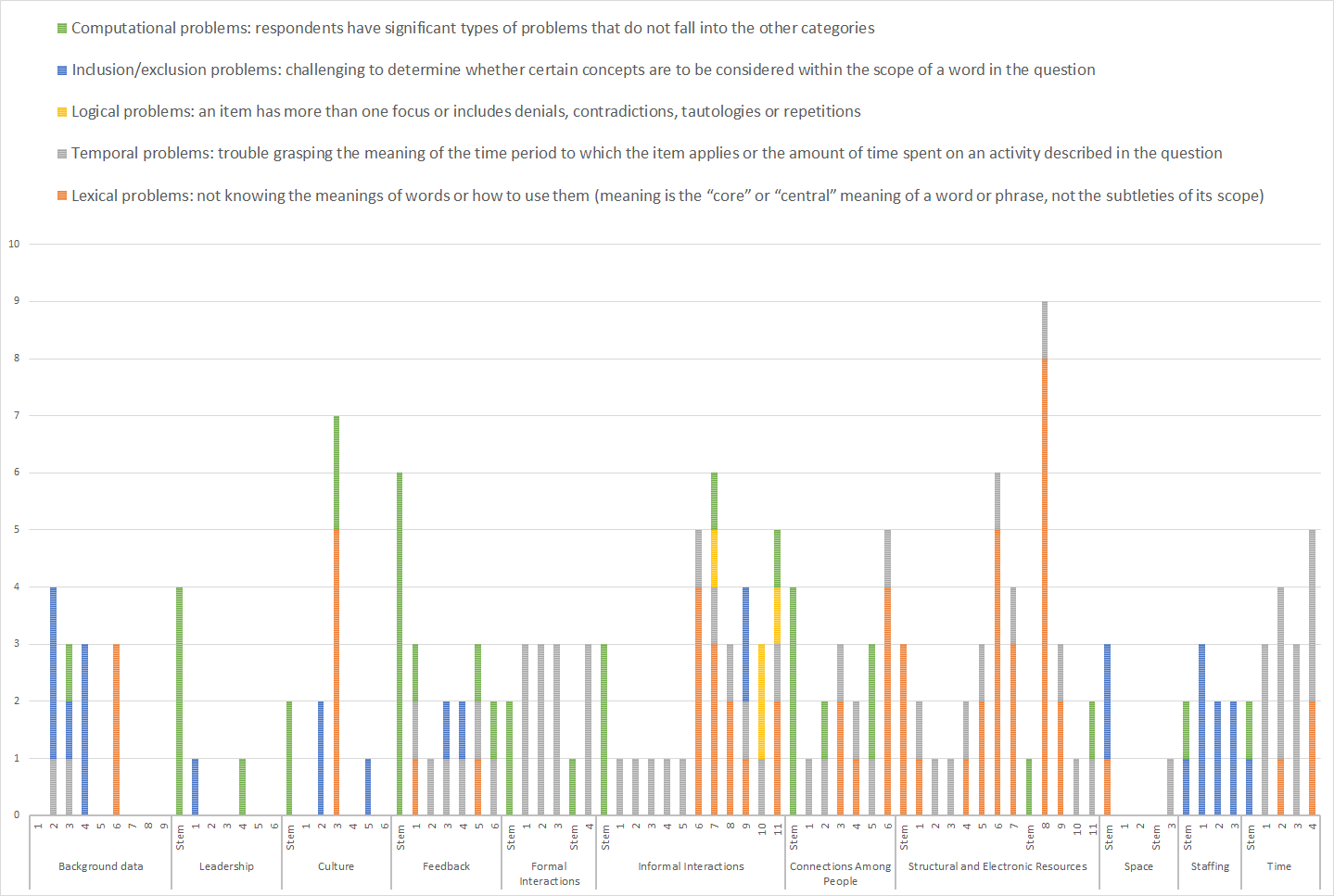
**

Supplement: S2 Appendix — (DOCX) [file pone.0258099.s002.docx]
