# Supplementary material for: Translation and validation of the Alberta Context Tool for use in Norwegian nursing homes
Source: PLoS One. 2021 Oct 8;16(10):e0258099. doi: 10.1371/journal.pone.0258099 (PMC8500415; doi:10.1371/journal.pone.0258099)
Supplement: S3 Appendix — (DOCX) [file pone.0258099.s003.docx]

**S3 Appendix 3. ACT translation.**

| Concept | Item | Min-max |
| --- | --- | --- |
| Lederskap | Ber om tilbakemelding selv når den kan være ubehagelig å høre. | 1-5 |
|  | Fokuserer på det man lykkes med heller enn det som mislykkes. | 1-5 |
|  | håndterer stressende situasjoner på en rolig måte. | 1-5 |
|  | Lytter aktivt, anerkjenner og responderer på henvendelser og bekymringer. | 1-5 |
|  | Veileder aktivt andre i deres arbeid. | 1-5 |
|  | Løser effektivt konflikter som oppstår. | 1-5 |
| Kultur | Jeg får anerkjennelse for jobben jeg gjør. | 1-5 |
|  | Jeg har kontroll på hvordan jeg gjør jobben min. | 1-5 |
|  | Organisasjonen min balanserer effektivt mellom anbefalt praksis fra forskningsbasert kunnskap og daglig drift. | 1-5 |
|  | Jeg får støtte til faglig utvikling. | 1-5 |
|  | Vi jobber for å gi beboerne og deres pårørende det de har behov for. | 1-5 |
|  | I min arbeidsgruppe støtter vi hverandre. | 1-5 |
| Tilbakemelding | Jeg får rutinemessig informasjon om hvordan min arbeidsgruppe presterer, med data som i eksemplene over (fall, smerte, tvang osv.). | 1-5 |
|  | Arbeidsgruppen vår har regelmessige uformelle diskusjoner om slike data. | 1-5 |
|  | Arbeidsgruppen vår har en formell rutine for å diskutere slike data. | 1-5 |
|  | Arbeidsgruppen vår lager rutinemessige tiltaksplaner på bakgrunn av slike data. | 1-5 |
|  | Arbeidsgruppen vår måler rutinemessig eget arbeid basert på disse tiltaksplanene. | 1-5 |
|  | Arbeidsgruppen vår sammenligner regelmessig egne resultater med andres. | 1-5 |
| Relasjoner | Kollegaer i gruppen deler informasjon med hverandre. | 1-5 |
|  | Mine observasjoner av beboernes tilstand blir rutinemessig tatt på alvor av de som har ansvar og myndighet. | 1-5 |
|  | Kollegaer i andre grupper deler informasjon med personer i min gruppe eller mitt team. | 1-5 |
|  | Jeg er komfortabel med å ta opp utfordringer vedrørende beboernes pleie- og omsorgstilbud med de som har ansvar og myndighet. | 1-5 |
|  | Målet med erfaringsutveksling innenfor og mellom grupper er å hjelpe andre å gjøre jobben sin. | 1-5 |
|  | Personer som deltar i arbeidsgruppen er verdsatt av andre i gruppen. | 1-5 |
| Bemanning | Vi har nok ansatte til å få gjort det nødvendige arbeidet. | 1-5 |
|  | Vi har nok ansatte til å gi best mulig pleie og omsorg. | 1-5 |
|  | Vi har nok ansatte for å sikre at beboerne har en så god dag som mulig. | 1-5 |
| Areal | Vi har tilfredsstillende plass til å gi beboerne pleie og omsorg. | 1-5 |
|  | Vi har plasser hvor vi kan sitte uforstyrret (for eksempel møterom) der vi kan diskutere og dele kunnskap og konfidensiell informasjon om beboere og pleieplaner. | 1-5 |
|  | Hvor ofte bruker du disse uforstyrrede plassene til å diskutere konfidensiell informasjon om beboerne, pleieplaner eller ny klinisk kunnskap? | 1-5 |
| Tid | Gjøre noe ekstra for beboerne? | 1-5 |
|  | Snakke med noen om pleieplaner for beboerne? | 1-5 |
|  | Slå opp noe (f.eks. i et tidsskrift, en bok eller på internett)? | 1-5 |
|  | Snakke med noen om ny klinisk kunnskap? | 1-5 |
| Uformell samhandling | Med sykepleier? | 1-5 |
|  | Med lege? | 1-5 |
|  | Med assistent? | 1-5 |
|  | Med helsefagarbeider eller hjelpepleier? | 1-5 |
|  | Med annet helsepersonell (utenom lege, sykepleier, helsefagarbeider eller assistent)? | 1-5 |
|  | Med forskningssykepleier? | 1-5 |
|  | Med praksislærer (fra skole)? | 1-5 |
|  | Med ansvarlig for kvalitetsforbedring? | 1-5 |
|  | Med noen som aktivt fremmer bruk av forskning i praksis? | 1-5 |
|  | Ved 'Korridorsamtaler' (f.eks. uformelle diskusjoner i gangen, på vaktrommet)? | 1-5 |
|  | Ved uformell opplæring i direkte pleiesituasjoner? | 1-5 |
|  | Med Fagsykepleier/sykepleiefaglig konsulent? | 1-5 |
| Formell samhandling | Teammøter om beboerne? | 1-5 |
|  | Tverrfaglig gjennomgang av beboerens pleie- og omsorgsbehov (gjennomgang av beboernes pleiebehov som helhet)? | 1-5 |
|  | Pårørendemøter (planleggingsmøter som involverer beboer og pårørende)? | 1-5 |
|  | Kompetanseheving (f.eks. konferanser, kurs, workshops) arrangert utenfor dette sykehjemmet? | 1-5 |
| Strukturelle og elektroniske ressurser | Et bibliotek? | 1-5 |
|  | Helsebiblioteket? | 1-5 |
|  | Lærebøker? | 1-5 |
|  | Tidsskrift (trykte/elektroniske)? | 1-5 |
|  | Oppslagstavler? | 1-5 |
|  | Strategiske dokumenter (trykte/elektroniske)? | 1-5 |
|  | Kliniske retningslinjer? | 1-5 |
|  | Fagprosedyrer? | 1-5 |
|  | Elektronisk beslutningsstøtte (datasystemer som støtte i pleie og beslutningstaking)? | 1-5 |
|  | Påminnelsessystem (f.eks. påminnelser via e-post)? | 1-5 |
|  | Nettside(r) på internett? | 1-5 |
|  | Internopplæring/workshops/kurs på dette sykehjemmet? | 1-5 |
