# Supplementary material for: Translation and validation of the Alberta Context Tool for use in Norwegian nursing homes
Source: PLoS One. 2021 Oct 8;16(10):e0258099. doi: 10.1371/journal.pone.0258099 (PMC8500415; doi:10.1371/journal.pone.0258099)
Supplement: S4 Appendix — (DOCX) [file pone.0258099.s004.docx]

**S4 Appendix 4. Standardized Factor Loadings and Model-Data Fit.**

|  | | **Factor Loadings** | | | | | | | |
| --- | --- | --- | --- | --- | --- | --- | --- | --- | --- |
| **Concept** | **Item** | **Model 1** | | **Model 2** | | | **Model 3** | | |
|  |  | **RN** | **LPN** | **RN** | **LPN** | | **RN** | | **LPN** |
| **Leadership** | Looks for feedback | 0.704 | 0.649 | 0.708 | | 0.656 |  |  | |
|  | Focuses on successes | 0.550 | 0.627 | 0.572 | | 0.639 |  |  | |
|  | Calmly handles stress | 0.746 | 0.775 | 0.730 | | 0.781 |  |  | |
|  | Listens, acknowledges, responds | 0.826 | 0.834 | 0.831 | | 0.821 |  |  | |
|  | Actively mentors and coaches | 0.823 | 0.847 | 0.823 | | 0.834 |  |  | |
|  | Resolves conflicts | 0.854 | 0.821 | 0.831 | | 0.817 |  |  | |
| **Culture** | Receive recognition | 0.698 | 0.741 | 0.675 | | 0.732 |  |  | |
|  | Control over work | 0.405 | 0.382 | 0.442 | | 0.401 |  |  | |
|  | Organization balances | 0.648 | 0.639 | 0.662 | | 0.619 |  |  | |
|  | Professional development | 0.657 | 0.677 | 0.665 | | 0.653 |  |  | |
|  | Clear on what patients want | 0.518 | 0.535 | 0.534 | | 0.548 |  |  | |
|  | Supportive work group | 0.583 | 0.555 | 0.592 | | 0.558 |  |  | |
| **Evaluation** | Routinely receive information | 0.745 | 0.768 | 0.751 | | 0.738 |  |  | |
|  | Discusses data informally | 0.646 | 0.675 | 0.660 | | 0.658 |  |  | |
|  | Formal process | 0.834 | 0.846 | 0.843 | | 0.846 |  |  | |
|  | Formulates action plans | 0.829 | 0.819 | 0.835 | | 0.808 |  |  | |
|  | Monitors our performance | 0.848 | 0.806 | 0.856 | | 0.805 |  |  | |
|  | Compares our performance | 0.687 | 0.566 | 0.689 | | 0.561 |  |  | |
| **Social Capital** | Share information with others | 0.693 | 0.702 | 0.691 | | 0.683 |  |  | |
|  | Observations are taken seriously | 0.666 | 0.728 | 0.661 | | 0.717 |  |  | |
|  | Information is shared | 0.487 | 0.438 | 0.504 | | 0.446 |  |  | |
|  | Comfortable talking in authority | 0.605 | 0.663 | 0.632 | | 0.660 |  |  | |
|  | Aim is to help others | 0.538 | 0.566 | 0.585 | | 0.580 |  |  | |
|  | Group participation is valued | 0.719 | 0.759 | 0.742 | | 0.734 |  |  | |
| **Slack – Staff** | Get the *necessary* work done | 0.762 | 0.739 | 0.754 | | 0.737 |  |  | |
|  | Deliver best possible care | 0.889 | 0.933 | 0.897 | | 0.920 |  |  | |
|  | Give patients a good day | 0.883 | 0.858 | 0.889 | | 0.878 |  |  | |
| **Slack – Space** | Adequate space | 0.563 | 0.624 | 0.569 | | 0.642 |  |  | |
|  | Private space | 0.957 | 0.917 | 0.956 | | 0.915 |  |  | |
|  | Use of private space | 0.733 | 0.615 | 0.748 | | 0.597 |  |  | |
| **Slack - Time** | Do something extra for patients | 0.709 | 0.678 | 0.721 | | 0.681 |  |  | |
|  | Talk about plan of care | 0.771 | 0.750 | 0.734 | | 0.737 |  |  | |
|  | Look something up | 0.733 | 0.694 | 0.753 | | 0.685 |  |  | |
|  | Talk about new clinical knowledge | 0.744 | 0.761 | 0.767 | | 0.723 |  |  | |
| **Informal Interactions** | Nurse | 0.850 | 0.687 |  | |  | 0.842 | 0.736 | |
|  | Physicians | 0.669 | 0.550 |  | |  | 0.665 | 0.539 | |
|  | Assistant | 0.795 | 0.714 |  | |  | 0.795 | 0.751 | |
|  | Allied health care provider | 0.881 | 0.760 |  | |  | 0.878 | 0.809 | |
|  | Other healthcare providers | 0.543 | 0.591 |  | |  | 0.531 | 0.538 | |
|  | Research nurse or coordinator | 0.149 | 0.356 |  | |  | 0.148 | 0.308 | |
|  | Clinical educator/instructor | 0.264 | 0.448 |  | |  | 0.288 | 0.411 | |
|  | Quality improvement representative | 0.344 | 0.599 |  | |  | 0.366 | 0.523 | |
|  | Champion | 0.207 | 0.440 |  | |  | 0.206 | 0.383 | |
|  | “Hallway talk” | 0.443 | 0.406 |  | |  | 0.451 | 0.432 | |
|  | Informal bedside teaching | 0.338 | 0.503 |  | |  | 0.354 | 0.492 | |
|  | Practice Development Nurse | 0.407 | 0.591 |  | |  | 0.421 | 0.551 | |
| **Formal Interactions** | Team meetings | 0.689 | 0.735 |  | |  | 0.702 | 0.754 | |
|  | Interdisciplinary meetings about patients | 0.835 | 0.844 |  | |  | 0.832 | 0.849 | |
|  | Family conferences/ next of kin meetings | 0.611 | 0.507 |  | |  | 0.599 | 0.562 | |
|  | Education out of workplace | 0.257 | 0.368 |  | |  | 0.255 | 0.343 | |
| **Structural/Electronic Resources** | Library | 0.059 | 0.212 |  | |  | 0.085 | 0.176 | |
|  | Health library (online) | 0.253 | 0.254 |  | |  | 0.278 | 0.241 | |
|  | Textbooks | 0.406 | 0.396 |  | |  | 0.444 | 0.382 | |
|  | Journals (printed/online) | 0.538 | 0.467 |  | |  | 0.509 | 0.481 | |
|  | Notice boards | 0.477 | 0.467 |  | |  | 0.497 | 0.480 | |
|  | Policies and procedures | 0.666 | 0.680 |  | |  | 0.674 | 0.678 | |
|  | Clinical practice guidelines | 0.752 | 0.729 |  | |  | 0.787 | 0.748 | |
|  | Professional procedures | 0.746 | 0.726 |  | |  | 0.794 | 0.758 | |
|  | Computerized decision support | 0.483 | 0.453 |  | |  | 0.462 | 0.431 | |
|  | Reminders (ex. via e-mail) | 0.541 | 0.532 |  | |  | 0.533 | 0.510 | |
|  | Websites | 0.534 | 0.560 |  | |  | 0.495 | 0.528 | |
|  | Internal training/workshops at work | 0.396 | 0.491 |  | |  | 0.404 | 0.460 | |
| **Model-Data Fit: x2 (p-value)** |  | 3273.669  (< .001) | 3856.242  (< .001) | 888.669  (< .001) | | 1115.927  (< .001) | 1263.213  (< .001) | 1781.037  (< .001) | |
|  |  | df = 1784 | df = 1784 | df = 506 | | df = 506 | df = 347 | df = 347 | |
| **RMSEA 2** |  | 0.052 | 0.056 | 0.047 | | 0.051 | 0.090 | 0.101 | |
| **SRMR 3** |  | 0.072 | 0.066 | 0.053 | | 0.052 | 0.093 | 0.097 | |
| **CFI 4** |  | 0.816 | 0.796 | 0.932 | | 0.916 | 0.708 | 0.657 | |

1 Horizontal lines separate factors within each model (i.e., there are 10 factors in Model 1, seven in Model 2 and three in Model 3)

2 RMSEA = Root Mean Square Error of Approximation

3 SRMR = Standardized Root Mean squared Residual

4 CFI = Comparative Fit Index
